# Trajectories and determinants of left ventricular ejection fraction after the first myocardial infarction in the current era of primary coronary interventions

**DOI:** 10.3389/fcvm.2022.1051995

**Published:** 2022-11-14

**Authors:** Peter Wohlfahrt, Dominik Jenča, Vojtěch Melenovský, Marek Šramko, Martin Kotrč, Michael Želízko, Jolana Mrázková, Věra Adámková, Jan Pitha, Josef Kautzner

**Affiliations:** ^1^Department of Preventive Cardiology, Institute for Clinical and Experimental Medicine (IKEM), Prague, Czechia; ^2^First Medical School, Charles University, Prague, Czechia; ^3^Department of Cardiology, Institute for Clinical and Experimental Medicine (IKEM), Prague, Czechia; ^4^Third Medical School, Charles University, Prague, Czechia; ^5^Medical and Dentistry School, Palacký University, Olomouc, Czechia

**Keywords:** myocardial infarction, ejection fraction (EF%), systolic dysfunction, inflammation, atrial fibrillation, epidemiology

## Abstract

**Background:**

Left ventricular ejection fraction (EF) is an independent predictor of adverse outcomes after myocardial infarction (MI). However, current data on trajectories and determinants of EF are scarce. The present study aimed to describe the epidemiology of EF after MI.

**Methods:**

Data from a single-center prospectively-designed registry of consecutive patients hospitalized at a large tertiary cardiology center were utilized.

**Results:**

Out of 1,593 patients in the registry, 1,065 were hospitalized for MI type I (65.4% STEMI) and had no previous history of heart failure or MI. At discharge, EF < 40% was present in 238 (22.3%), EF 40–50% in 326 (30.6%) and EF > 50% in 501 (47.0%). Patients with EF < 40% were often those who suffered subacute and anterior STEMI, had higher heart rate at admission and higher maximal troponin level, and had more often HF signs requiring intravenous diuretics. Among subjects with EF < 40%, the follow-up EF was available in 166 (80% of eligible). Systolic function recovered to EF > 50% in 39 (23.1%), slightly improved to EF 40–50% in 44 (26.0%) and remained below 40% in 86 (50.9%). Systolic function improvement to EF > 40% was predicted by lower severity of coronary atherosclerosis, lower leukocyte count, and the absence of atrial fibrillation.

**Conclusions:**

Despite recent improvements in in-hospital MI care, one in five patients has systolic dysfunction at hospital discharge. Out of these, EF improves in 51%, and full recovery is observed in 23%. The severity of coronary atherosclerosis, inflammatory response to MI, and atrial fibrillation may affect EF recovery.

## Graphical Abstract

Trajectories and determinants of EF after the first MI. 
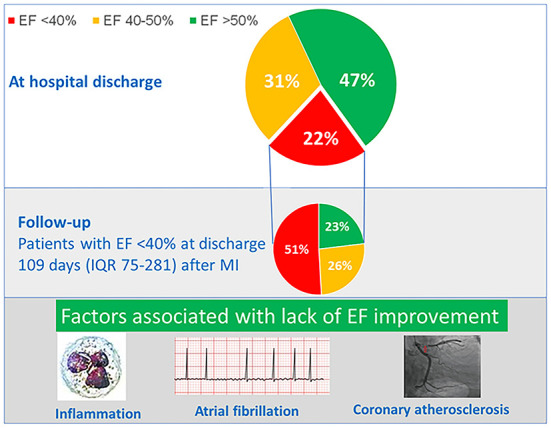

## Introduction

Left ventricular ejection fraction (EF) is a guideline-recommended tool for risk stratification of patients with acute myocardial infarction (MI) (1). Numerous studies have shown that low EF after MI is associated with an increased risk of cardiovascular and total mortality, heart failure, and sudden cardiac death (2–5). Several studies have also shown that EF may improve after hospital discharge, and such EF recovery is associated with a lower risk of cardiovascular events (6–9) and improved quality of life (10). The phenotype of heart failure with improved ejection fraction has been recently recognized by the guidelines and refers to patients with previous heart failure with reduced ejection fraction who have an LVEF > 40% (11, 12).

In the last 20 years, the implementation of evidence-based therapy as primary percutaneous coronary intervention (PCI), dual antiplatelet therapy, and statin therapy have significantly improved MI mortality (13, 14). This may have also influenced systolic dysfunction prevalence and trajectories after MI. However, epidemiologic studies evaluating systolic dysfunction prevalence and trajectories coming from the contemporary era of MI therapy are scarce. Therefore, we sought to evaluate the incidence, trajectories, and determinants of left ventricular ejection fraction among consecutive patients hospitalized for their first MI.

## Methods

### Population

This study utilized data from the prospective AMBITION registry (Institute for Clinical and Experimental Medicine Acute Myocardial Infarction Registry), which collects clinical data and biospecimens from all consecutive patients hospitalized for acute coronary syndrome at a tertiary heart center since June 2017. During the hospital stay, all patients underwent detailed interviews, and additional information was obtained through manual chart abstraction and laboratory studies. For this analysis, data from individuals without previous history of heart failure and coronary artery disease, hospitalized for type I MI between June 2017 and November 2021 were used. The institutional review board of the Institute for Clinical and Experimental Medicine approved the study, and all participants signed informed consent.

### Left ventricular ejection fraction

Left ventricular EF was measured using transthoracic echocardiography. In patients with several in-hospital EF measurements, the last one before hospital discharge was used as the baseline value. According to baseline EF, patients were categorized as having systolic dysfunction (EF < 40%), mid-range EF (EF 40–50%), or preserved systolic function (EF > 50%). In patients with systolic dysfunction at the time of MI hospitalization, optimal medical therapy with angiotensin converting enzyme inhibitor, beta-blocker and spironolactone was initiated. However, in patients with contraindications as hypotension or bradycardia/bradyarrhythmia this was not initiated. The patient was discharged with the recommendation for OMT therapy up-titration by an outpatient cardiologist. In patients with EF < 40% at hospital discharge, follow-up EF beyond 6 weeks from the index hospitalization was recorded. By the follow-up EF, patients with systolic dysfunction at hospital discharge were categorized as having full EF recovery (follow-up EF > 50%), slightly improved EF (follow-up EF 40–50%), or persistent systolic dysfunction (follow-up EF < 40%).

### Definition of comorbidities

History of diabetes was defined by the use of oral antidiabetic drugs or insulin at the time of hospital admission or by glycated hemoglobin ≥48 mmol/L at the time of hospitalization. Arterial hypertension was defined as self-reported use of antihypertensive drugs at admission. Self-reported history of smoking was used. A person was considered a current smoker if smoking at least one cigarette per day during the last 12 months. Positive family history of CVD was defined by MI or stroke in the first-degree relatives before 55 years in males and before 60 years in females, respectively.

Coronary artery stenosis degree was based on percent diameter stenosis by visual estimation done by an experienced invasive cardiologist. Culprit lesion intervention was performed during the index hospitalization. In patients with multiple vessel disease, additional interventions of non-culprit lesions were done during the index-hospitalization or patients were invited for additional elective procedure, aiming for complete revascularization. During the follow-up, in none of the studied patient additional intervention was required due to restenosis, in-stent thrombosis or recurrent MI.

Gensini score was used to quantify the overall severity of coronary artery atherosclerosis, while accounting for lesion location, as previously described (15, 16). Mortality data were provided by the Institute of Health Information and Statistics, keeping a list of all deceased by law.

### Statistical methods

Data are presented as mean ± standard deviation, median (interquartile range–IQR), or frequency (percent). Analysis of variance (ANOVA), Kruskal-Wallis or chi-square tests were used to compare differences across the three EF groups, as appropriate. Multivariate logistic and linear regression were used to assess factors associated with systolic dysfunction at baseline and EF recovery at follow-up. Factors with a significant association (p < 0.05) in the univariate analysis (Table 1; Supplementary Table 1) were used as inputs for the multivariate model. Log-rank test was used to compare survival by EF categories. Cox proportional hazard model was used to assess the prognostic value of EF.

**Table 1:** Population demographics by left ventricular ejection fraction at the time of hospitalization.

**Variable**	**EF < 40**	**EF 40–50**	**EF > 50**	***p* for linear trend**
	***N =* 238**	***N =* 326**	***N =* 501**	
Age, years	66.2 ± 12.6	62.8 ± 12.2	63.4 ± 11.7	0.012
Male gender, *n* (%)	177 (74.4)	249 (76.4)	368 (73.5)	0.654
**Risk factors**				
Arterial hypertension, *n* (%)	106 (44.7)	145 (44.5)	193 (38.6)	0.074
Diabetes, *n* (%)	65 (27.3)	84 (25.8)	122 (24.4)	0.380
Current smoking, *n* (%)	101 (42.4)	168 (51.5)	218 (43.5)	0.801
Statin use before admission, *n* (%)	35 (14.7)	46 (14.1)	112 (22.4)	0.003
Family history of CVD, *n* (%)	68 (28.6)	75 (23.0)	151 (30.1)	0.371
COPD, *n* (%)	15 (6.3)	22 (6.7)	26 (5.2)	0.457
AF history, *n* (%)	11 (4.6)	15 (4.6)	24 (4.8)	0.905
**Index event**				
CPR before admission, *n* (%)	19 (8.0)	14 (4.3)	20 (4.0)	0.032
STEMI, *n* (%)	202 (84.9)	252 (77.3)	242 (48.5)	0.0001
Subacute MI, *n* (%)	63 (26.5)	51 (15.6)	35 (7.0)	0.0001
Killip class >1, *n* (%)	114 (47.9)	59 (18.1)	47 (9.4)	0.0001
Selective coronarography, *n* (%)	233 (97.9)	323 (99.1)	500 (99.8)	0.009
PCI, *n* (%)	196 (82.4)	277 (85.0)	434 (86.6)	0.129
CABG, *n* (%)	17 (7.1)	35 (10.7)	43 (8.6)	0.732
In-hospital AF, *n* (%)	44 (18.5)	46 (14.1)	40 (8.0)	0.0001
Pericarditis, *n* (%)	14 (5.9)	7 (2.1)	6 (1.2)	0.0001
Intravenous diuretics, *n* (%)	135 (56.7)	64 (19.6)	53 (10.6)	0.0001
Anterior MI, *n* (%)	186 (78.2)	134 (41.1)	142 (28.3)	0.0001
Admission SBP, mmHg	138.2 ± 25.4	140.4 ± 26.3	147.5 ± 26.8	0.0001
Admission DBP, mmHg	79.9 ± 15.9	78.5 ± 14.1	79.6 ± 12.5	0.958
Admission heart rate, min^−1^	85.2 ± 20.2	76.8 ± 16.8	73.9 ± 16.4	0.0001
Max Troponin natural log, ng/L	7.58 ± 1.56	7.47 ± 1.30	6.4 ± 1.4	0.0001
CKD EPI, ml/min/1.73 m^2^	73.9 ± 23.2	78.5 ± 22.1	78.6 ± 21.3	0.014
BMI, kg/m^2^	28.3 ± 4.8	28.6 ± 4.9	28.9 ± 4.9	0.135
HbA1c, mmol/L/mol	45.9 ± 13.3	45.7 ± 13.9	44.5 ± 12.4	0.145
Fasting glycemia, mmol/L	9.4 ± 4.6	8.4 ± 3.9	7.8 ± 3.2	0.0001
Total cholesterol, mmol/L	4.9 ± 1.3	4.9 ± 1.2	4.8 ± 1.2	0.829
Triglycerides, mmol/L	1.7 ± 1.7	1.6 ± 1.0	1.9 ± 1.3	0.048
HDL cholesterol, mmol/L	1.2 ± 0.3	1.2 ± 0.3	1.1 ± 0.3	0.002
LDL cholesterol, mmol/L	3.2 ± 1.1	3.3 ± 1.1	3.2 ± 1.1	0.998
Leukocytes, 10^9^/L	12.4 ± 4.3	12.0 ± 4.0	11.3 ± 20.7	0.309
Erythrocytes, 10^12^/L	4.7 ± 0.6	4.7 ± 0.5	4.6 ± 0.5	0.683
Hemoglobin, g/L	141.9 ± 16.8	142.8 ± 16.7	142.2 ± 14.6	0.939
**Discharge medication**				
ACEi/ARB, *n* (%)	163 (72.4)	258 (79.4)	377 (75.9)	0.538
Beta-blocker, *n* (%)	187 (83.1)	279 (85.8)	384 (77.3)	0.017
Statin, *n* (%)	209 (92.9)	314 (96.6)	485 (97.6)	0.003
Furosemide, *n* (%)	143 (63.6)	56 (17.2)	30 (6.0)	<0.001
Spironolactone, *n* (%)	157 (69.8)	45 (13.8)	16 (3.2)	<0.001
Acetylsalicylic acid, *n* (%)	208 (92.4)	306 (94.2)	481 (96.8)	0.009
Clopidogrel, *n* (%)	87 (38.7)	83 (25.5)	132 (26.6)	0.004
Prasugrel, *n* (%)	5 (2.2)	5 (1.5)	16 (3.2)	0.285
Ticagrelor, *n* (%)	119 (52.9)	222 (68.3)	335 (67.4)	0.001
Warfarin, *n* (%)	24 (10.7)	29 (8.9)	17 (3.4)	<0.001
Apixaban, *n* (%)	4 (1.8)	3 (0.9)	3 (0.6)	0.147
Dabigatran, *n* (%)	6 (2.7)	6 (1.8)	4 (0.8)	0.049
Rivaroxaban, *n* (%)	5 (2.2)	5 (1.5)	8 (1.6)	0.614
**Outcome**				
Death, *n* (%)	39 (16.4)	18 (5.5)	32 (6.4)	0.0001

## Results

Of 1,593 patients in the AMBITION registry, 1,347 had type I MI. Of these, 268 had a previous history of coronary artery disease, and another 14 had chronic heart failure history. Of the 1,065 eligible patients (65.4% STEMI), all had available EF at the time of MI hospitalization.

### Systolic dysfunction at the time of MI hospitalization

**Figure 1:**
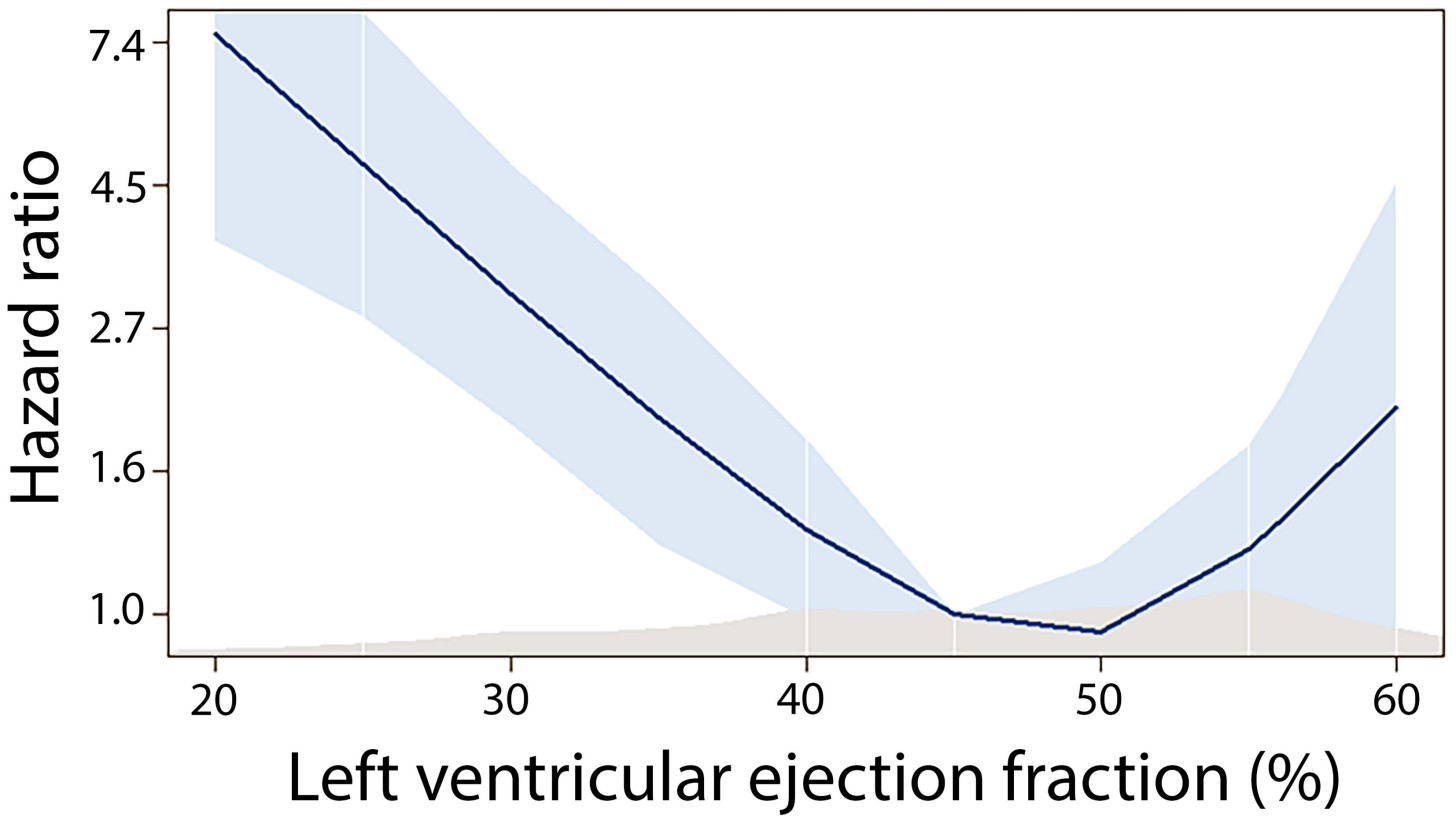
Association between discharge ejection fraction and mortality risk.

Baseline echocardiography was performed on the median 1 day (IQR 0–2) after MI. Systolic dysfunction with EF below 40% was present in 238 (22.3%), mid-range systolic function with EF 40–50% in 326 (30.6%) and EF above 50% in 501 (47.0%), respectively. Population demographics by EF categories are shown in Table 1. In the multivariate analysis (Table 2), patients with systolic dysfunction at the time of hospitalization (EF < 40%) were more likely to experience subacute and anterior STEMI, had higher heart rate at admission and higher maximal troponin level, more often clinical signs of heart failure requiring intravenous diuretic therapy and more often pericarditis. After adjustment for age and gender, we found a non-linear association between discharge EF and mortality risk, with increased mortality in subjects with EF < 40% (Figure 1). In the multivariate model, discharge EF was an independent predictor of total mortality risk after MI (Table 3).

**Table 2:** Multivariate logistic regression of factors associated with EF < 40% at the time of hospitalization.

**Variable**	**OR (95% CI)**	***p***
Anterior MI	8.39 (5.57–12.65)	0.001
Admission heart rate	1.01 (1.00–10.2)	0.01
STEMI	2.57 (1.60–4.14)	0.001
Subacute MI	1.95 (1.20–3.20)	0.01
Pericarditis	3.13 (1.12–8.74)	0.029
Intravenous diuretics	3.64 (2.16–6.13)	0.001
Maximal troponin level	1.22 (1.07–1.40)	0.003
Killip class above I	2.00 (1.15–3.45)	0.013

**Table 3:** Cox regression of factors associated with mortality after myocardial infarction.

**Variable**	**HR (95% CI)**	***p***
Age	1.047 (1.021–1.74)	0.001
CKD EPI	0.978 (0.968–0.989)	0.001
Current smoking	1.875 (1.153–3.048)	0.011
LV EF		0.004
EF < 40% vs. EF > 50%	1.841 (1.065–3.184)	0.029
EF 40–50% vs. EF > 50%	0.669 (0.357–1.251)	0.208
AF during hospitalization	1.688 (1.024–2.785)	0.040
Glycemia	1.063 (1.019–1.110)	0.005
Killip class >I	2.339 (1.402–3.900)	0.001
STEMI	0.510 (0.322–0.809)	0.004

### Recovery of systolic function

Of the 238 patients with EF < 40% at the time of hospitalization, follow-up EF was not available in 26 due to in-hospital death or death within 6 months since the hospital discharge. Of the 212 eligible patients, follow-up EF was collected in 169 (80% of eligible). The follow-up systolic function evaluation was done on a median of 109 days (IQR 75–281) after MI. During this period, systolic function recovered to EF > 50% in 39 (23.1%), slightly improved to EF 40–50% in 44 (26.0%) and remained below 40% in 86 (50.9%).

**Figure 2:**
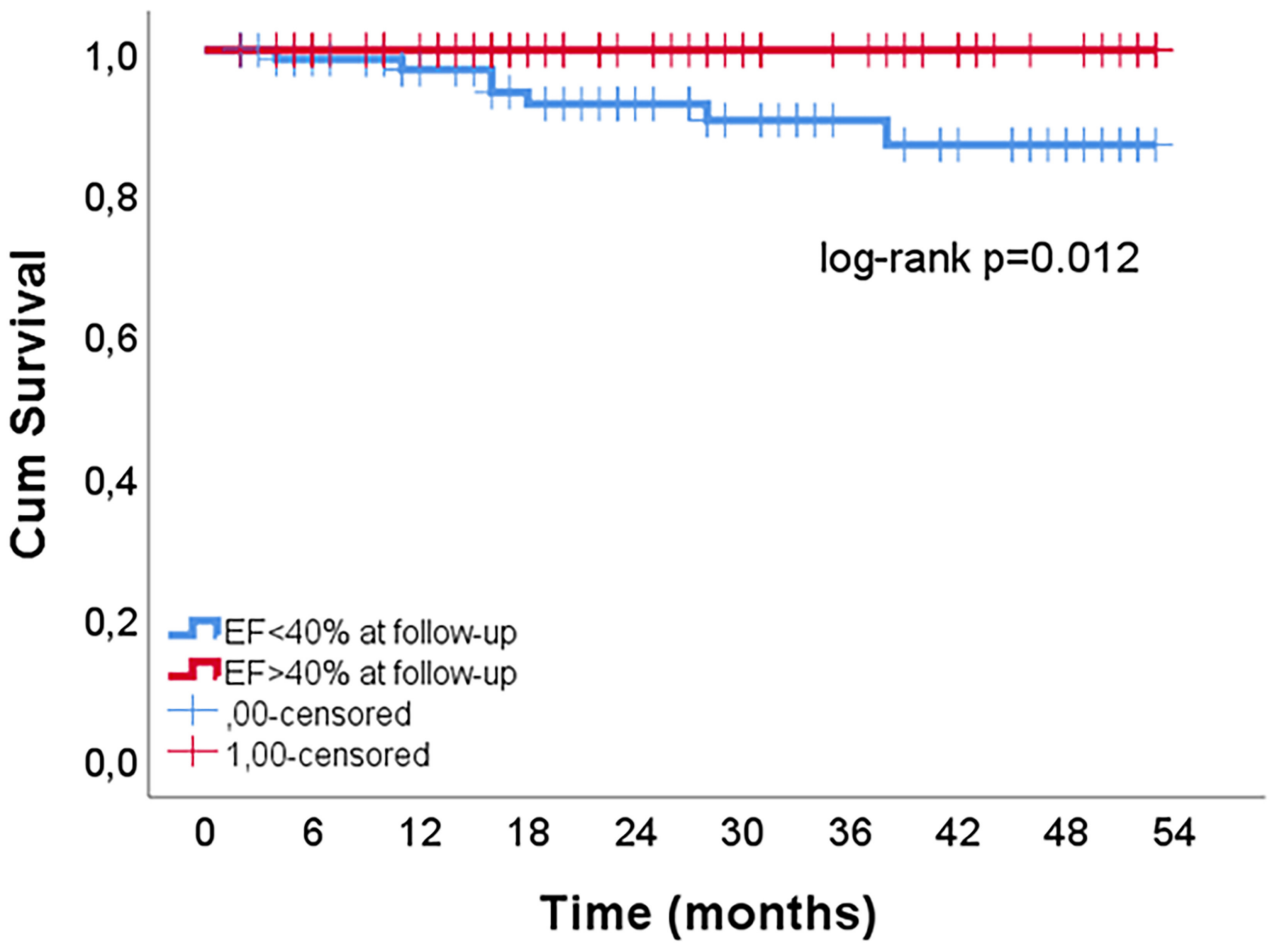
The influence of EF improvement on mortality risk in patients with EF<40% at hospital discharge.

Characteristics of patients by EF improvement at follow-up are shown in the Supplementary Table 1. In the multivariate analysis, improvement in systolic function to EF > 40% was predicted by lower severity of coronary artery atherosclerosis (lower GENSINI score), a higher discharge EF, lower leukocyte count and the absence of atrial fibrillation (AF) during MI hospitalization (Table 4). These factors were confirmed in the sensitivity analysis with the absolute change in EF as a dependent variable, with the addition of female gender associated with EF improvement (Supplementary Table 2). Recovery of systolic function was associated with lower mortality risk (log-rank *p* = 0.012) (Figure 2).

**Table 4:** Multivariate logistic regression of factors associated with systolic function improvement to EF to >40% during follow-up.

**Variable**	**OR (95% CI)**	***p***
Coronary atherosclerosis severity (GENSINI score)	0.983 (0.969–0.997)	0.017
Leukocyte count	0.827 (0.735–0.931)	0.002
AF during hospitalization	0.359 (0.130–0.995)	0.049
Left ventricular ejection fraction	1.212 (1.100–1.337)	0.001

## Discussion

The present study shows that in the current era of MI therapy, one in five patients after the first MI has reduced EF. In the months following the MI, one in four patients will fully recover EF, with severity of coronary atherosclerosis, inflammatory response, and AF being associated with lack of EF improvement.

There is a lack of historical data on left ventricular systolic function after MI, because EF was not routinely measured in the past. In the Euro Heart Survey analyzing MI management in the year 2000 in 25 European countries, only 73% of STEMI and 61% of non-STEMI patients had EF measured (17). Thus, reported data may be a subject of a selection bias. This may bias direct comparison of historical data coming from the thrombolysis era with data observed in our study.

In the present study, 53% of patients at hospital discharge had EF < 50%. This is very similar to the 46–60% prevalence observed in the thrombolysis era (18–20). Similarly, the 22% prevalence of EF < 40% in our study is close to the 27–36% range observed at the turn of the century (21–24). Thus, despite significant improvements in MI management, systolic dysfunction immediately after MI is still common, with a prevalence similar to that observed in the thrombolysis era. There are several explanations for this finding. First, recent improvements in pre-hospital care led to a decrease in out of hospital mortality (25, 26). Second, introduction of PCI has decreased in-hospital mortality (27, 28). Thus, more patients that would previous die pre- or in-hospital are discharged with systolic dysfunction. Third, the landscape of MI patients is changing, (29) with risk factors as obesity and obesity-related comorbidities increasing especially in young patients with MI (30). Therefore, the higher burden of metabolic risk factors may have influenced systolic dysfunction prevalence.

Among patients with EF < 40% at the hospital discharge, we have observed full EF recovery in 23%. This is much lower than the 42% EF recovery rate observed in a retrospective cohort study of consecutive young patients aged ≤ 50 hospitalized for their first MI (9). While we did not find any direct effect of age on EF recovery in the present study, the different burden of comorbidities affecting EF recovery in younger subjects may explain this difference. On the other hand, the observed 51% proportion of patients with systolic function improvement to EF ≥ 40 in the present study is higher than the 24% proportion observed at the turn of the century (24). In other recent studies, the proportion of patients with systolic function improvement varies around 50% (8, 31, 32). This suggests that implementation of evidence-based therapy may have increased the proportion of patients with EF recovery. Recent recommendation to use Sodium-glucose Cotransporter-2 (SGLT2) inhibitors and angiotensin receptor-neprilysin inhibitor (ARNI) in patients with heart failure with reduced ejection fraction may further increase the proportion of patients with EF improvement after MI (11). However, the PARADISE-MI study in patients with acute MI did not show superiority of ARNI on cardiovascular mortality and incident heart failure as compared to ramipril (33).

In the present study, we have identified several factors that may influence the course of EF recovery. Increased leukocytes count as a proxy of excess innate immunity activation was associated with a lower likelihood of EF improvement at follow-up. Lately, the importance of inflammation in patients after MI has been increasingly recognized (34). Due to excessive and prolonged inflammatory response to MI leukocytes infiltrate viable border zone of the infarction, thereby extending ischemic injury beyond the original MI zone (35). Prolonged inflammation also triggers adverse left ventricular remodeling. In the CANTOS study among patients after MI, monoclonal antibody targeting IL-1β significantly reduced recurrent major adverse cardiovascular events (36). Similarly, a low-dose colchicine, a potent anti-inflammatory drug affecting inflammasome, has decreased risk of ischemic cardiovascular events in patients after recent MI (37). Our results suggest that targeting of the inflammatory resolution pathways may influence EF recovery in patients with systolic dysfunction and increased inflammatory response to MI.

Another factor identified in the present study, that increased mortality risk by 69% and decreased EF improvement odds by 64%, was the new onset of AF during the MI hospitalization. On the other hand, pre-existing AF was not associated with mortality risk or EF recovery. This is in line with a previous study, in which mortality risk associated with a new onset AF during MI was 87% higher as compared to pre-existing AF (38). Several mechanisms such as atrial ischemia, volume overload, inflammation, and pericarditis have been described to trigger AF during MI (39). Thus new onset AF may be a marker of risk factors, which are known to affect EF recovery and increase heart failure risk. However direct hemodynamic effects of AF caused by the loss of atrial contraction, heart rate irregularity and increased heart rate may negatively influence EF recovery (40). Whether targeting patients with new onset AF can decrease mortality risk and improve EF after MI needs to be further evaluated.

In our sensitivity analysis, female gender was associated with a higher increase in EF during follow-up. This is in line with a meta-analysis of 18 studies, in which females had a higher odds of EF recovery (41). The gender difference may be explained by a higher level of signaling molecules with anti-inflammatory effects and more reparative immune cells in females (42).

Our study evaluating EF trajectories after MI is limited by the echocardiographic method of EF measurement. A large intra- and inter-individual variability in echocardiographic EF measurement has been reported (43). Despite this limitation, our and other studies have shown the prognostic value of this parameter. Because follow-up EF was available in 80% of eligible patients, our results may be influenced by the selection bias. The major strength of our study is the use of prospective registry which collects data of all consecutive patients hospitalized for MI at a high-volume center. This precludes several sources of bias. Furthermore, all patient records were adjudicated by the study physicians, which is more accurate that data derived from billing codes.

In summary, systolic dysfunction after the first MI is still common, with 1 in 5 patients having EF < 40%. Severity of coronary atherosclerosis, inflammatory response to MI, and AF may all affect EF recovery. These observations provide novel therapeutic targets for EF recovery.

## Data availability statement

The raw data supporting the conclusions of this article will be made available by the authors, upon reasonable request.

## Ethics statement

The studies involving human participants were reviewed and approved by IKEM Ethics Committee. The patients/participants provided their written informed consent to participate in this study.

## Author contributions

All authors have contributed in full extent to the conception and design of the work, to the acquisition of data and/or their analysis, interpretation, have participated in drafting the manuscript, revising it critically for its intellectual content, and have given their approval of the final version to be published.

## Funding

Supported by Ministry of Health of the Czech Republic, Grant nr. NV 19-09-00125 and by the project National Institute for Research of Metabolic and Cardiovascular Diseases (Programme EXCELES, Project No. LX22NPO5104)—Funded by the European Union—Next Generation EU.

## Conflict of interest

PW has received consulting fees or honoraria from Servier. JK reports grants and personal fees from Biosense Webster, Biotronik, Boston Scientific, Medtronic, grants and personal fees from Abbott (SJM), personal fees from Merit Medical, Daiichi Sankyo, Boehringer Ingelheim, BMS, Bayer, Merck, MSD, Pfizer, all outside the submitted work. The remaining authors declare that the research was conducted in the absence of any commercial or financial relationships that could be construed as a potential conflict of interest.

## Publisher's note

All claims expressed in this article are solely those of the authors and do not necessarily represent those of their affiliated organizations, or those of the publisher, the editors and the reviewers. Any product that may be evaluated in this article, or claim that may be made by its manufacturer, is not guaranteed or endorsed by the publisher.
