# Supplementary material for: Trajectories and determinants of left ventricular ejection fraction after the first myocardial infarction in the current era of primary coronary interventions
Source: Front Cardiovasc Med. 2022 Nov 14;9:1051995. doi: 10.3389/fcvm.2022.1051995 (PMC9702523; doi:10.3389/fcvm.2022.1051995)
Supplement: Supplementary file 1 [file Data_Sheet_1.docx]

**Supplementary table 1** Comparisson of patients with EF<40 at hospital discharge by improvement to EF ≥40

| **Variable** | **No improvement**  N=85 | **Improvement**  N=84 | **p** |
| --- | --- | --- | --- |
| Age, years | 65.3±10.9 | 61.9±12.3 | 0.06 |
| Male gender, n (%) | 69 (81.2) | 63 (75.0) | 0.357 |
| **Risk factors** |  |  |  |
| Arterial hypertension, n (%) | 45 (52.9) | 40 (47.6) | 0.540 |
| Diabetes, n (%) | 23 (27.1) | 23 (27.4) | 1.00 |
| Current smoking, n (%) | 39 (45.9) | 38 (45.2) | 1.00 |
| Family history of CVD, n (%) | 29 (34.1) | 26 (31.0) | 0.743 |
| COPD, n (%) | 6 (7.1) | 3 (3.6) | 0.496 |
| AF history, n (%) | 6 (7.1) | 3 (3.6) | 0.496 |
| **Index event** |  |  |  |
| CPR before admission, n (%) | 8 (9.4) | 7 (8.3) | 1.00 |
| STEMI, n (%) | 73 (85.9) | 72 (85.7) | 1.0 |
| Subacute MI, n (%) | 24 (28.2) | 17 (20.2) | 0.282 |
| Killip class >1, n (%) | 46 (54.1) | 26 (31.0) | 0.003 |
| Selective coronarography, n (%) | 84 (98.8) | 83 (98.8) | 1.00 |
| PCI, n (%) | 75 (88.2) | 72 (85.7) | 0.655 |
| CABG, n (%) | 2 (2.4) | 7 (8.3) | 0.099 |
| In-hospital AF, n (%) | 20 (23.5) | 8 (9.5) | 0.022 |
| Pericarditis, n (%) | 7 (8.2) | 3 (3.6) | 0.329 |
| Intravenous diuretics, n (%) | 57 (67.1) | 30 (35.7) | 0.0001 |
| Anterior MI, n (%) | 68 (80.0) | 70 (83.3) | 0.692 |
| Admission SBP, mmHg | 136.9±22.5 | 144.0±26.8 | 0.063 |
| Admission DBP, mmHg | 81.5±13.4 | 82.2±18.0 | 0.750 |
| Admission heart rate, min^-1^ | 90.5±21.4 | 81.5±16.5 | 0.003 |
| Max Troponin natural log, ng/L | 7.65±1.81 | 7.38±1.75 | 0.291 |
| CKD EPI, ml/min/1.73 m² | 77.0±19.5 | 80.0±21.3 | 0.348 |
| BMI, kg/m^2^ | 28.7±4.6 | 32.1±3.7 | 0.770 |
| HbA1c, mmol/L/mol | 45.6±11.8 | 47.1±16.3 | 0.514 |
| Glycemia, mmol/L | 9.4±4.2 | 9.3±4.6 | 0.878 |
| Total cholesterol, mmol/L | 5.1±1.5 | 4.9±1.1 | 0.377 |
| Triglycerides, mmol/L | 1.4 [1.02-2.02] | 1.34 [1.03-2.03] | 0.961 |
| HDL cholesterol, mmol/L | 1.2±0.3 | 1.1±0.3 | 0.255 |
| LDL cholesterol, mmol/L | 3.3±1.3 | 3.3±1.1 | 0.805 |
| Leukocytes, 10^9^/L | 13.3±3.9 | 11.5±3.8 | 0.002 |
| Erythrocytes, 10^12^/L | 4.7±0.5 | 4.8±0.6 | 0.099 |
| Haemoglobin, g/L | 142.1±14.1 | 146.2±16.2 | 0.081 |
| GENSINI score | 74.9±41 | 53.8±24.1 | 0.0001 |
| EF at discharge, % | 28.1±4.8 | 32.1±3.7 | 0.0001 |
| **Outcome** |  |  |  |
| Death, n(%) | 7 (8.2) | 0 (0.0) | 0.014 |

**Supplementary table 2** Multivariate linear regression of factors associated with systolic function change during follow-up

| **Variable** | **Beta** | **p** |
| --- | --- | --- |
| Leukocyte count logarithm | -8.116 | <0.0001 |
| GENSINI score logarithm | -3.121 | 0.009 |
| Female gender | 3.446 | 0.030 |
| AF during hospitalization | -3.536 | 0.043 |

Negative values of beta coeficients mean decrease in follow-up EF
